# Supplementary material for: Limitations of Tamoxifen Application for In Vivo Genome Editing Using Cre/ERT2 System
Source: Int J Mol Sci. 2022 Nov 15;23(22):14077. doi: 10.3390/ijms232214077 (PMC9694728; doi:10.3390/ijms232214077)
Supplement: Supplementary file 1 [file ijms-23-14077-s001.zip › ijms-2003326-supplementary.pdf]

Supplementary Materials

Table S1. Averaged substance concentrations and their standard deviations after administration of a single 3 mg dose.

Organ	Time	Administ-ration	Tam, ng/g	Endo, ng/g	OH-Tam, ng/g	Tam range	Endo range	OH-Tam range
uterus	4	fed	2108.57	28.20	924.40	1350.38-3346.81	19.51-43.5	600.07-1547.04
uterus	4	injected	486637.8	743.15	11898.46	383741.86-605256.33	428.94-1034.67	10850.04-12670.11
uterus	17	fed	1300.33	56.40	415.30	175.87-2356.26	15.82-85.46	0-952.97
uterus	17	injected	215627.4	5038.46	47908.45	26964.14-404290.66	2668.78-7408.15	5453.83-90363.07
liver	4	fed	5371.96	242.81	4786.52	1764.08-10636.76	80.56-332.87	1855.68-7989.26
liver	4	injected	409681.3	5054.06	50652.93	186070.36-699973.62	2455.38-6829.67	21140.02-74729.23
liver	17	fed	3632.36	318.17	2322.52	2942.09-4065.07	154.84-431.26	1534.04-3459.56
liver	17	injected	26265.46	2810.80	12759.49	1428.31-40136.75	436.05-4156.9	693.86-19498.02
lung	4	fed	1774.53	15981.9	20655.34	714.49-3116.83	6770.92-22200.92	7991.96-34301.2
lung	4	injected	7037.82	219905.1	58575.05	5833.89-8865.69	188612.32-253902.54	56313.12-60652.4
lung	17	fed	5656.16	17281.81	22117.68	2080.64-8560.93	11222.32-28839.14	10131.21-44220
lung	17	injected	35605.4	214646.1	123605.7	14988.68-61565.04	32013.93-328653.97	19529.95-202549.42
brain	4	fed	1258.56	8.66	936.47	838.03-1679.08	8.37-8.94	758.4-1114.54
brain	4	injected	42738.53	146.85	11622.35	26486.17-58704.72	95.08-201.36	7154.29-13951.3
brain	17	fed	2483.82	28.48	814.17	1719.75-3273.51	22.6-32.33	399-1292.95

brain	17	injected	23625.87	183.64	6644.77	3052.23-41214.27	47.22-285.31	420.71-9860.4

Table S2. Primers used for genotyping.

Primer name	Primer sequence	Annealing temperature
Cdk8-F	5′-CTAATTGGAGTCCTGGACAAGGC-3′	58°C
Cdk8-R2	5′-AGCGTAGTCTTTATCGTCCTTC-3′	58°C
Cdk8-R	5′-CCCATAGAGCCATCTCAACAGC-3′	58°C
